# Edoxaban eliminates hypercoagulability evoked by transient temperature changes in human whole blood

**DOI:** 10.1002/joa3.12945

**Published:** 2023-11-06

**Authors:** Anna Suzuki, Satomi Hamada, Ai Oono, Yuki Hasegawa, Yasuteru Yamauchi, Kaoru Okishige, Kenzo Hirao, Tetsuo Sasano

**Affiliations:** ^1^ Department of Cardiovascular Medicine Tokyo Medical and Dental University (TMDU) Tokyo Japan; ^2^ Department of Clinical Laboratory Tokyo Medical and Dental University (TMDU) Hospital Tokyo Japan; ^3^ Department of Cardiology, Japan Redcross Yokohama City Bay Hospital Yokohama Japan; ^4^ Yokohama Minato Heart Clinic Yokohama Japan; ^5^ AOI International Hospital Kawasaki Japan

**Keywords:** atrial fibrillation, catheter ablation, coagulability, dielectric blood coagulometry, factor Xa inhibitor

## Abstract

**Background:**

Thrombosis is a common critical complication relating to radiofrequency catheter ablation and cryoablation. There is a possibility that high‐temperature stimulation during radiofrequency ablation or low‐temperature stimulation during cryoablation may affect the coagulability of blood. In this study, we aimed to determine the impacts of transient temperature stimulations on the coagulability of whole blood and to clarify if edoxaban suppressed the hypercoagulability.

**Methods:**

Citrated blood samples were drawn from 41 healthy subjects. Some blood samples were mixed with tissue factor (TF) and several concentrations of edoxaban (50, 100, and 200 ng/mL). Blood samples were exposed to several temperature stimulations for 1 min: heat stimulation (50°C) or cryostimulation (−20°C), and compared with control (37°C). Repeated cryostimulations or sequential cryo‐ and heat stimulation were also applied. Coagulability of whole blood was measured using a dielectric blood coagulometry. As an index of coagulability, the end of acceleration time (EAT) was used.

**Results:**

Both heat‐ and cryostimulations significantly shortened the EAT compared to the control, indicating that hypercoagulability was induced by temperature stimulations. Application of TF enhanced and extended the hypercoagulability after the temperature stimulations. Sequential application of cryo‐ followed by heat stimulation further enhanced the hypercoagulability of blood. Application of edoxaban increased the EAT in a concentration‐dependent manner in control condition. Edoxaban at 100 or 200 ng/mL completely suppressed the shortening of EAT evoked by these temperature stimulations.

**Conclusion:**

Transient temperature stimulations evoked hypercoagulability regardless of cryo‐ or heat stimulation. Edoxaban with 100 ng/mL or more eliminated this temperature‐stimulated hypercoagulability.

## Graphical abstract

(Upper) End of acceleration time (EAT), an indicator of whole blood coagulability, was shortened after heat‐ or cryoapplications, indicating temperature change induced hypercoagulability. (Lower) Administration of edoxaban at 100 ng/mL diminished the hypercoagulability induced by temperature stimulation.

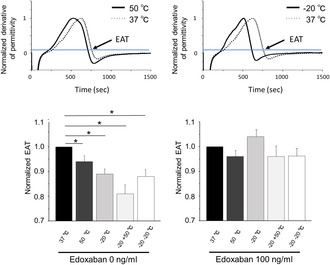

## 1 INTRODUCTION

Atrial fibrillation (AF) is the most common sustained form of tachyarrhythmias and an independent risk factor for stroke.^1^ Considering the Virchow's triad, AF is accompanied with the reduction of blood flow and the impairment of endocardial cells. Thus, the hypercoagulability strongly contributed to the thrombus formation in the settings of AF. Therefore, anticoagulant therapy with direct oral anticoagulants (DOACs) or warfarin is required to prevent stroke in patients with AF.

Pulmonary vein (PV) isolation by catheter ablation is widely used as a therapeutic option for AF to restore sinus rhythm. To date, there are mainly two types of ablation procedures classified by energy source: radiofrequency ablation and cryoablation. Radiofrequency ablation achieves isolation between pulmonary veins and atrium by creating contiguous transmural lesions by the point‐by‐point focal radiofrequency energy delivery, while cryoablation creates circumferential transmural lesions through balloon‐based technology, which offers a homogeneous freezing area and thus results in irreversible ablation in a single application. Both energy sources are sometimes used in combination in case if the cryoballoon application fails to complete PV isolation.

Some of the serious complications of catheter ablation procedure are periprocedural stroke, transient ischemic attack (TIA), and asymptomatic cerebral emboli.^2^ Periprocedural thromboembolic events typically occur during the first 24 h after catheter ablation, although the incidence of stroke varies largely between studies (0%–5%).^3^ Aagaard P et al.^3^ summarized that large thromboembolic events mainly relate to dislodgement of pre‐formed thrombus, and that the mechanisms of asymptomatic cerebral emboli remain unclear, but may include small thrombi, tissue, char, and/or air emboli. The activation of the coagulation cascade would contribute to the formation of periprocedural thrombi. In fact, the increases in coagulation markers, prothrombin fragment F1 + 2, and D‐dimer, were observed during the periprocedural period of both radiofrequency ablation and cryoablation.^4^, ^5^ Thus, to avoid thromboembolic events, uninterrupted anticoagulation by oral anticoagulants such as warfarin or direct oral anticoagulant (DOACs) and maintenance of an adequate activated clotting time by heparin during the procedure appear useful.^6^, ^7^, ^8^, ^9^, ^10^, ^11^

There are two potential mechanisms of the activation of the coagulation cascade during catheter ablation procedures. One is the contact of blood to artificial surfaces like sheaths or ablation catheters inserted into the left atrium, and the other is the exposure of blood to tissue factor on damaged tissues caused by catheter ablation. In addition, there is a possibility that high‐temperature stimulation during radiofrequency ablation or low‐temperature stimulation during cryoablation applied to blood might affect the coagulation. However, the impacts of these transient temperature changes induced by catheter ablation on the coagulability were not evaluated so far.

Recently, we have developed a novel dielectric blood coagulometry (DBCM) for the evaluation of whole blood coagulability.^12^, ^13^, ^14^, ^15^ DBCM measures the temporal change in whole blood dielectric permittivity, which mainly represents the aggregation and deformation of red blood cells. A novel index, the end of acceleration time (EAT), represents the whole blood coagulability and has the potential to detect small changes in the hypercoagulable state. A study in patients with AF showed that the coagulability of whole blood obtained from AF patients was enhanced.^16^

In this study, we determined the changes in whole blood coagulability using DBCM after whole blood was given a temporal temperature changes that mimicked radiofrequency ablation and cryoablation. Moreover, we investigated the effect of one of DOACs, edoxaban,^17^ on hypercoagulable state.

## 2 METHODS

### 2.1 Study subjects

The study group consisted of 41 healthy subjects (23.2 ± 0.9 years old, 19 males). The healthy subjects were defined as having no medical history, no medications, no family history of coagulation deficiency, and no abnormal bleeding events. Another 11 subjects (seven AF patients and four controls) were enrolled for the measurement of tissue factor. This study was approved by the ethics committee of Tokyo Medical and Dental University (No. 2000‐1849, and No. 2000‐1534). Blood samples were collected after written informed consent.

### 2.2 Blood collection and preparation of anticoagulated samples

Blood samples were drawn from the cubital vein with minimum stasis. The first 0.5 mL of the drawn blood was discarded, and the remaining blood was kept in a tube containing 3.13% sodium citrate. The blood was kept at 37°C with or without 0.3 pg/mL tissue factor reagent (R&D systems, Minneapolis, MN, USA). To measure plasma tissue factor (TF) concentration in AF patients, plasma samples obtained from AF patients during catheter ablation.

Edoxaban was supplied by Daiichi Sankyo Co., Ltd. (Tokyo, Japan). Edoxaban was dissolved in 100% DMSO to make a 180 mM stock solution, diluted with distilled water, and added to the whole blood samples to obtain final concentrations of 0, 50, 100, and 200 ng/mL. The edoxaban concentrations were decided within its plasma concentration range at the standard dose of 60 mg, peak plasma concentration was 302 ng/mL.^18^

### 2.3 Dielectric blood coagulometry

Whole blood coagulability was assessed using a prototype dielectric coagulometer (Sony Corp., Tokyo, Japan) as previously described.^16^ Briefly, 180 μL of citrated whole blood was initially mixed with 15 μL of 160 mM CaCl_2_ (final concentration of Ca^2+^ was 12.0 mM). The blood sample was incubated at 37°C throughout the measurement. The DBCM measured the dielectric permittivity in a frequency range from 100 Hz to 16 MHz, with a sampling interval of 1 min. The dielectric permittivity was normalized compared to its initial value and represented as normalized permittivity. The result of the DBCM was analyzed by conducting a 5‐point derivative of the dielectric permittivity at 10 MHz.^16^ As an index for whole blood coagulability, the end of acceleration time (EAT) was determined. EAT was defined as the time to 10% of the maximum value of permittivity in the descending phase.

### 2.4 Heat‐ or cryostimulation of whole blood

**FIGURE 1:**
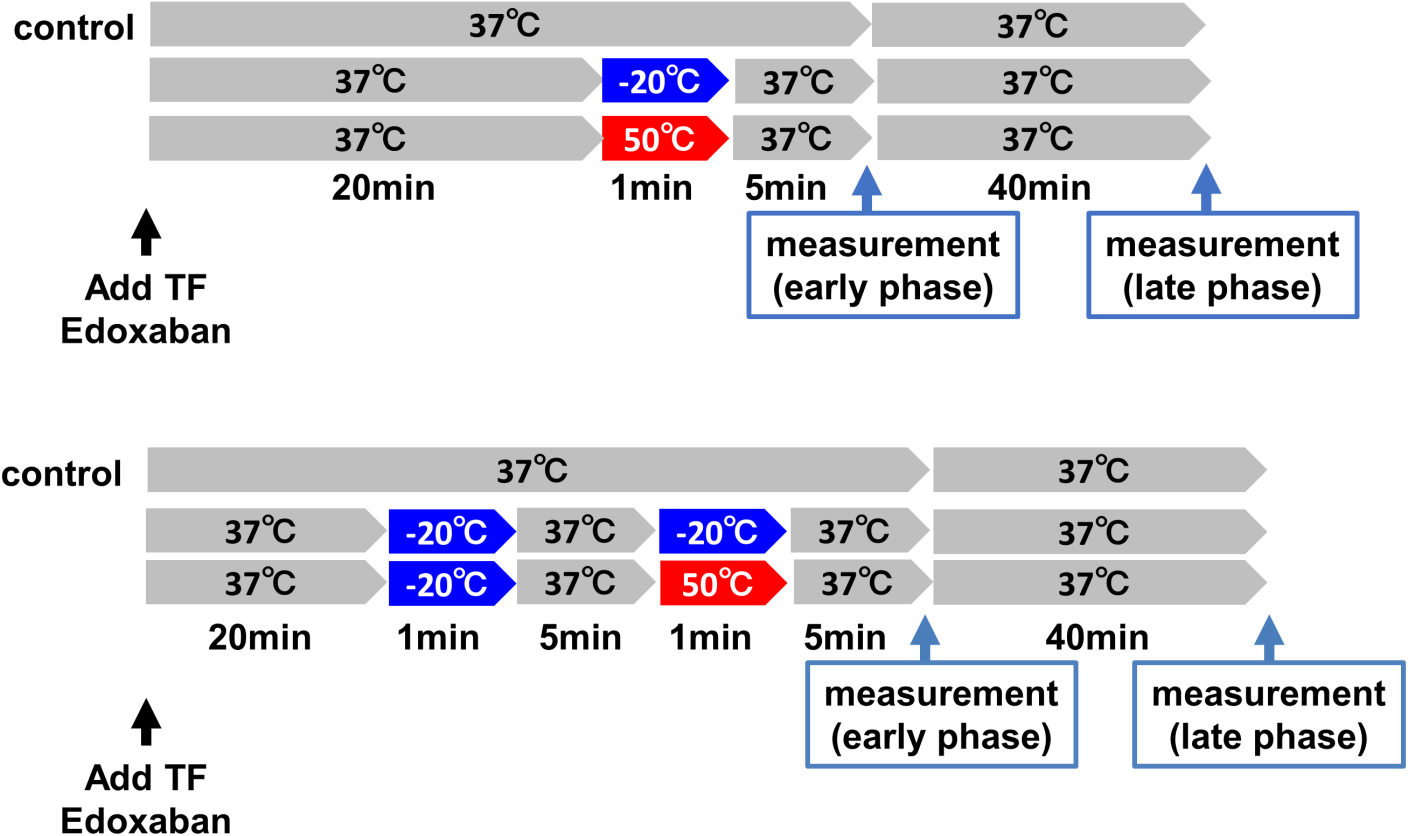
The schematic protocol of temperature stimulation on blood sample and measurements of whole blood coagulability. In a single temperature‐stimulation protocol (upper panel), heat‐ (50°C) or cryo‐ (−20°C) stimulations were applied for 1 min. The measurement of whole blood coagulability was done at 5 min after the cessation of temperature stimulation (early phase), and 45 min after the temperature stimulation (late phase). In a repeated or sequential temperature stimulation protocols (lower panel), two temperature stimulations were applied with 5 min of interval. The time point for the measurement of coagulability was identical to that of single stimulation protocol.

In a single stimulation study, heat stimulation (50°C) or cryostimulation (−20°C) was applied to whole blood for 1 min using an incubator or a freezer. In a repeated stimulation study, repeated cryostimulation (−20°C and −20°C) or sequential cryo‐ and heatstimulation (−20°C and 50°C) was applied for 1 min each with 5 min of interval (Figure 1). We chose 1 min for heat‐ or cryostimulation because application of cryostimulation for longer duration resulted in the formation of ice, which affected the measurement by DBCM.

### 2.5 Study protocol

After stabilization of whole blood samples at 37°C for 20 min, the blood were stimulated with 50°C or −20°C, and the temperature was back to 37°C. Since a preliminary study indicated that the blood temperature returned to 37°C within 5 min after transient heat‐ or cryostimulations, DBCM analysis was performed 5 min after the transient temperature changes (early phase). The EAT was normalized to that of the control sample which was kept at 37°C and expressed as relative shortening. Then, the temperature was kept at 37°C for following 40 min, and second measurement was done (late phase) (Figure 1).

In sequential temperature stimulation protocol, we applied temperature stimulation with an interval of 5 min. Considering the clinical application of touch‐up ablation, the sequential protocol was set at −20°C or 50°C after first application of −20°C (Figure 1).

### 2.6 Measurement of tissue factor

For the measurement of tissue factor, the blood samples were collected with EDTA‐2Na tube. Then, the sample was centrifuged at 1690 x g  for 5 min to extract plasma. The concentration of tissue factor was measured using Human TF ELISA Kit (Assaypro LLC, St. Charles, MO, USA), and plate reader (Bio‐Rad Laboratories, Inc., Model 680, Hercules, CA, USA) according to the manufacturers instrument.

### 2.7 Statistical analysis

Statistical analyses were performed with JMP®10 software (SAS Institute Inc., Cary, NC, USA). Data are expressed as mean ± standard error of mean. The statistically significant difference was evaluated by Student's *t*‐test or ANOVA. Multiple comparisons were evaluated by Dunnett test. A *p* < .05 was considered statistically significant.

## 3 RESULTS

### 3.1 Impacts of heat‐ and cryostimulations on whole blood coagulability

**FIGURE 2:**
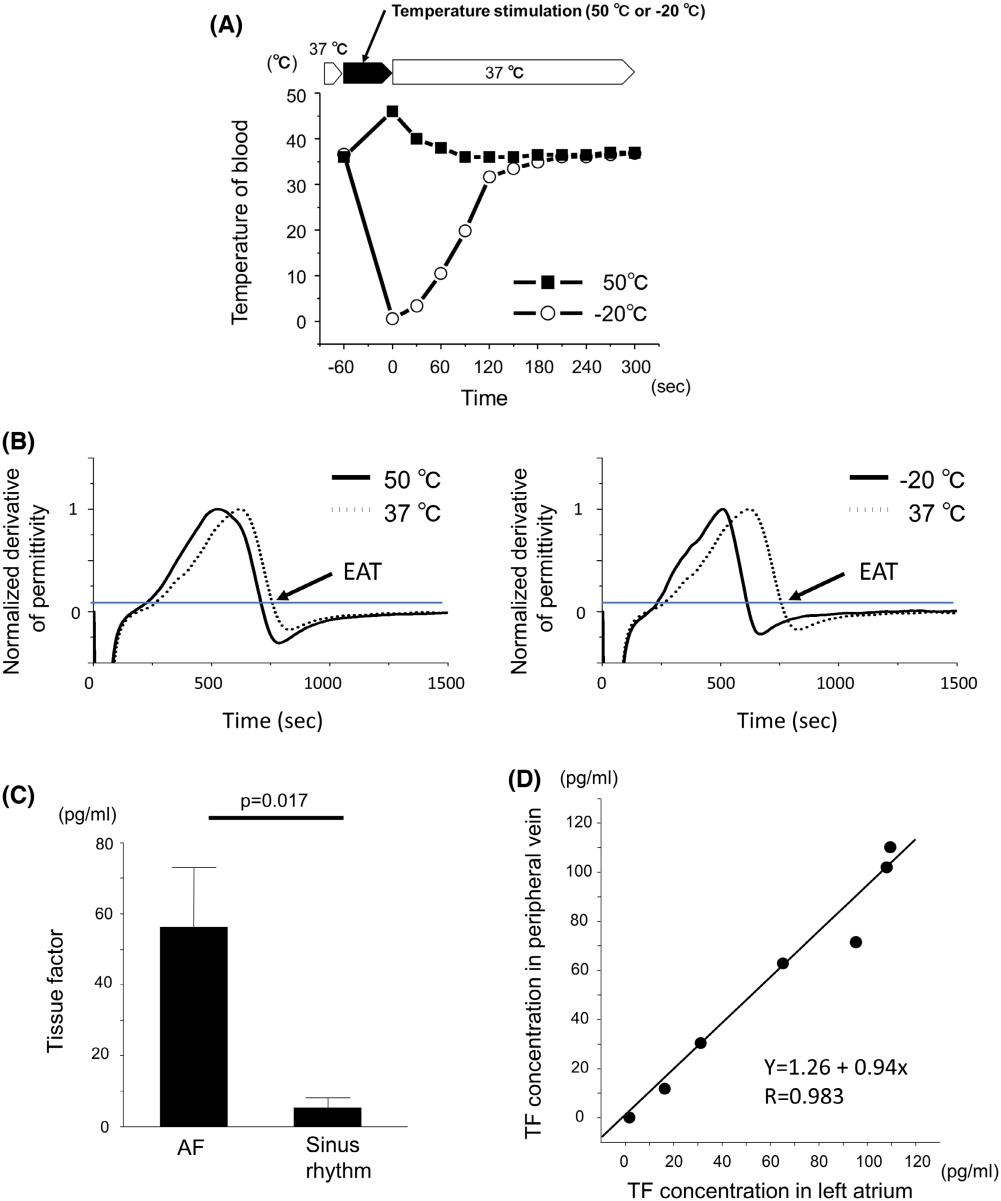
Serial temperature changes of blood sample and its coagulability after heat‐ and cryostimulations. (A) The temperature of blood sample was serially measured after the application of heat‐ (50°C) or cryo‐ (−20°C) stimulations for 1 min. The temperature of blood samples were measured every 30 s after the stimulations. After 180 s, the temperature of blood returned to 37°C. (B) Representative curves of the normalized derivative of permittivity in blood treated with transient temperature stimulations with 50°C or −20°C. The end of acceleration time, defined with the time to reach 10% of the maximum value in the descending phase was shortened by both temperature stimulations. (C) Comparison of the plasma concentration of tissue factor in subjects with or without AF. (D) Correlation of the tissue factor concentration in left atrium and peripheral blood in a same patient. They show linear correlation (y = 1.26 + 0.94x, *R* = .983, *p* < .001).

Figure 2A shows the changes in temperature of blood after single heatstimulation (50°C) or cryostimulation (−20°C) for 1 min. The blood temperature changed temporarily and returned to 37°C within 5 min after transient heat‐ or cryostimulations. Figure 2B shows the representative traces of permittivity after temporal temperature changes. We calculated the derivative of normalized permittivity and defined EAT (end of acceleration time) representing whole blood coagulation.^16^ Both heat‐ and cryostimulations shortened EAT, indicating the acceleration of coagulability.

We then measured the tissue factor (TF) concentration in plasma obtained from AF patients during catheter ablation and normal control. The plasma concentration of TF was significantly higher in patients with AF compared to those with sinus rhythm (Figure 2C). We also measured the TF concentration in patients underwent catheter ablation and compared the TF level between the blood obtained in left atrium (LA) and peripheral vein (Figure 2D). These findings indicated that TF concentration was increased similarly in patients with AF at LA during catheter ablation procedure.

**FIGURE 3:**
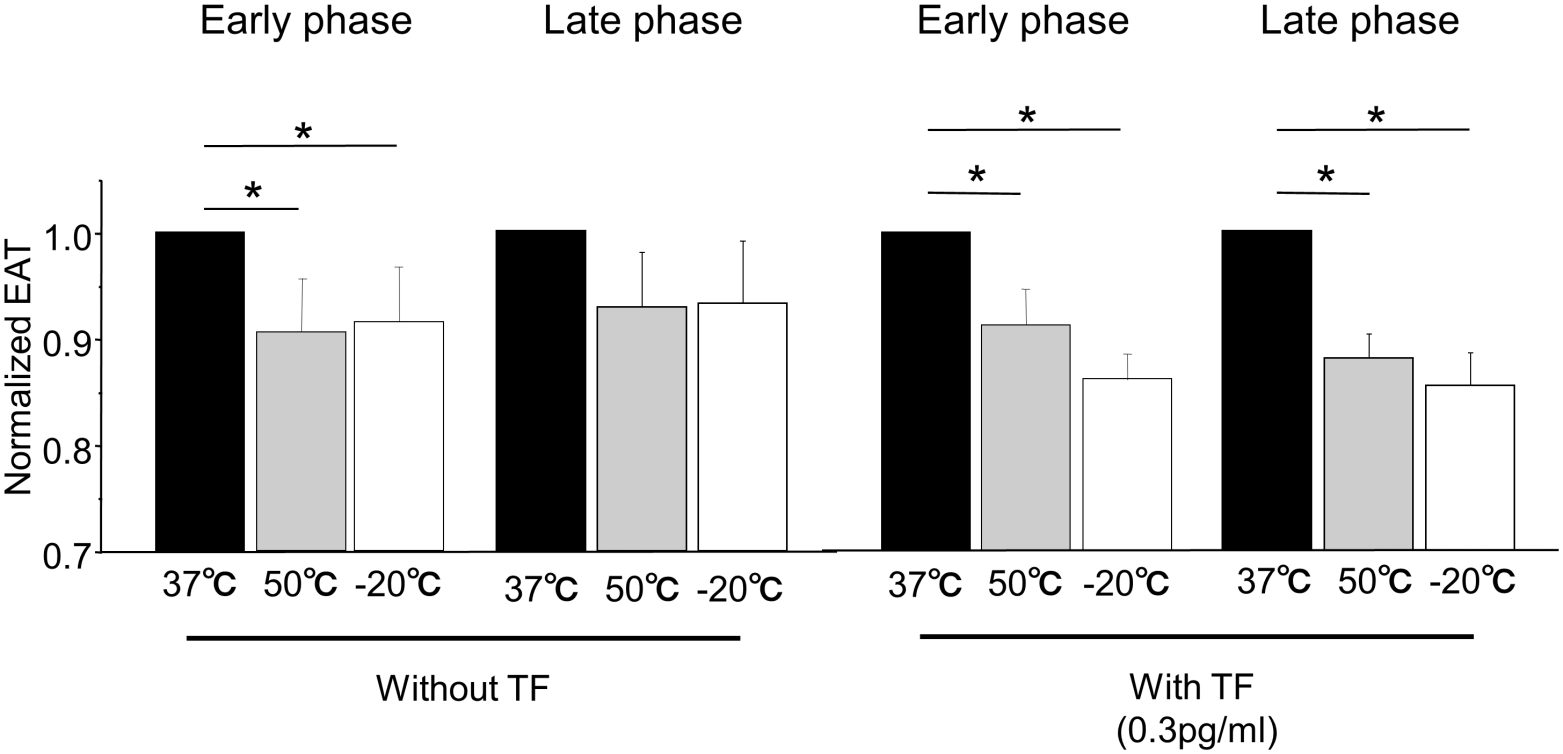
Impacts of the single application of heat‐ and cryostimulations on whole blood coagulability. (Left) Comparison of the end of acceleration time (EAT) among blood samples treated with heat‐ (50°C) or cryo‐ (−20°C) stimulations or control (37°C) in the absence of TF. (Right) In the presence of TF, the shortening of EAT tended to be accelerated. Of note, the EAT at late phase was significantly shorter in both heat‐ and cryostimulations compared to the control, indicating the presence of TF prolonged the increased coagulability. *p < .05.

At first, we performed single application of heat‐ or cryo‐temperature stimulation. As shown in Figure 1, we measured blood coagulability at 5 min after the temperature stimulation (early phase), and 40 min after the early phase measurement (late phase). Both heat‐ (50°C) or cryo‐ (−20°C) stimulations significantly shortened the EAT in early phase, indicating that transient temperature changes induced hypercoagulability in human whole blood. However, the change did not reach the significant difference in late phase (Figure 3, left). The addition of TF enhanced the hypercoagulability in the cryostimulated blood compared with those without TF at early phase (13.9% ± 2.8% vs. 8.4% ± 2.4% shortening of EAT, respectively, *p* = .024). The application of TF also enhanced the hypercoagulability by heat‐ and cryostimulations at late phase, which showed significant shortening of EAT compared to the control, which indicated the hypercoagulability was sustained at least for additional 40 min in the presence of TF (Figure 3, right).

**FIGURE 4:**
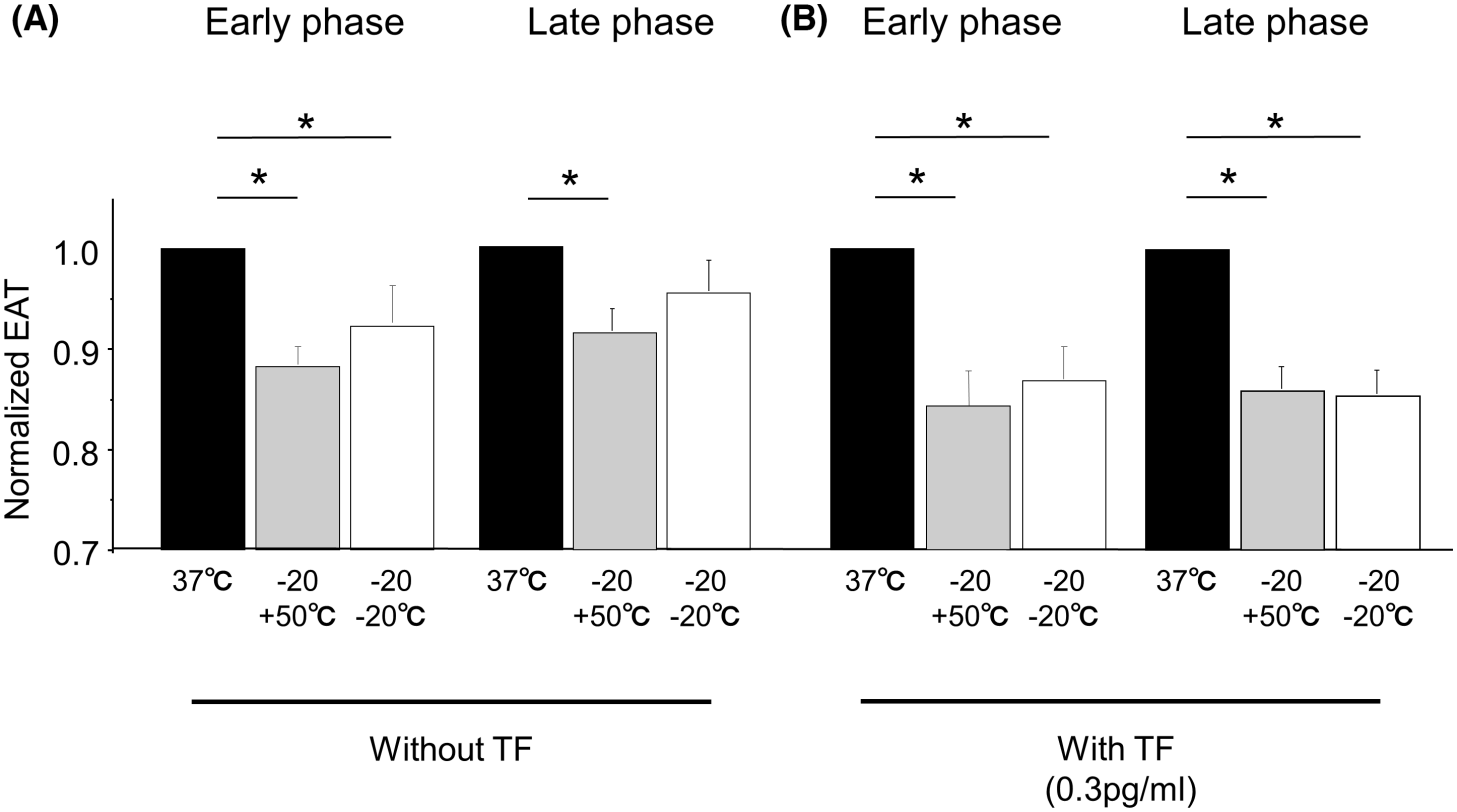
Impacts of the repeated cryostimulations and the sequential cryo‐ and heatstimulation on whole blood coagulability. (A) Comparison of the EAT among blood samples treated with repeated (−20 + −20°C) and sequential (−20 + 50°C) stimulations or control (37°C) in the absence of TF. (B) In the presence of TF. *p < .05.

We then applied repeated cryostimulations or the sequential cryo‐ and heatstimulation. Both stimulations induced shortening of the EAT in early phase (7.8% ± 3.4% and 12.1% ± 1.5% shortening, respectively) (Figure 4, left). Addition of TF enhanced the blood coagulability in both stimulations (13.1% ± 3.0% and 15.6% ± 3.6% shortening of EAT by repeated and sequential stimulations in early phase, respectively). And the increased coagulability sustained for additional 40 min, indicated by the shortening of EAT in late phase (14.2% ± 2.4% and 12.6% ± 2.2% shortening, respectively).

### 3.2 Effect of edoxaban on hypercoagulability induced by transient temperature changes

**FIGURE 5:**
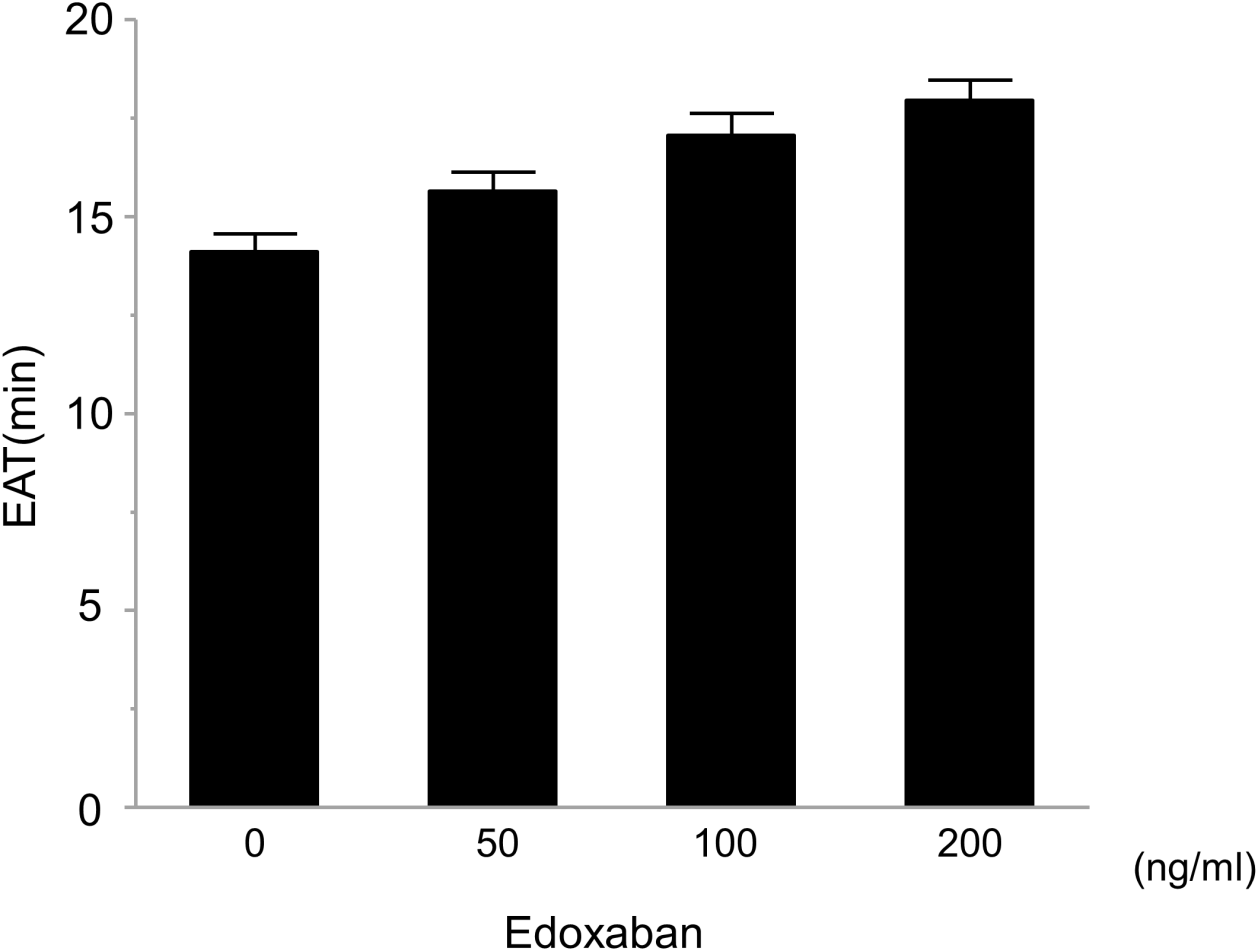
Effect of edoxaban on the end of acceleration time in human whole blood. Edoxaban (50–200 ng/mL) was added to human whole blood, and the end of acceleration time (EAT) was determined. Edoxaban showed the increase in EAT in a dose‐dependent manner (*p* < .01 by ANOVA).

**FIGURE 6:**
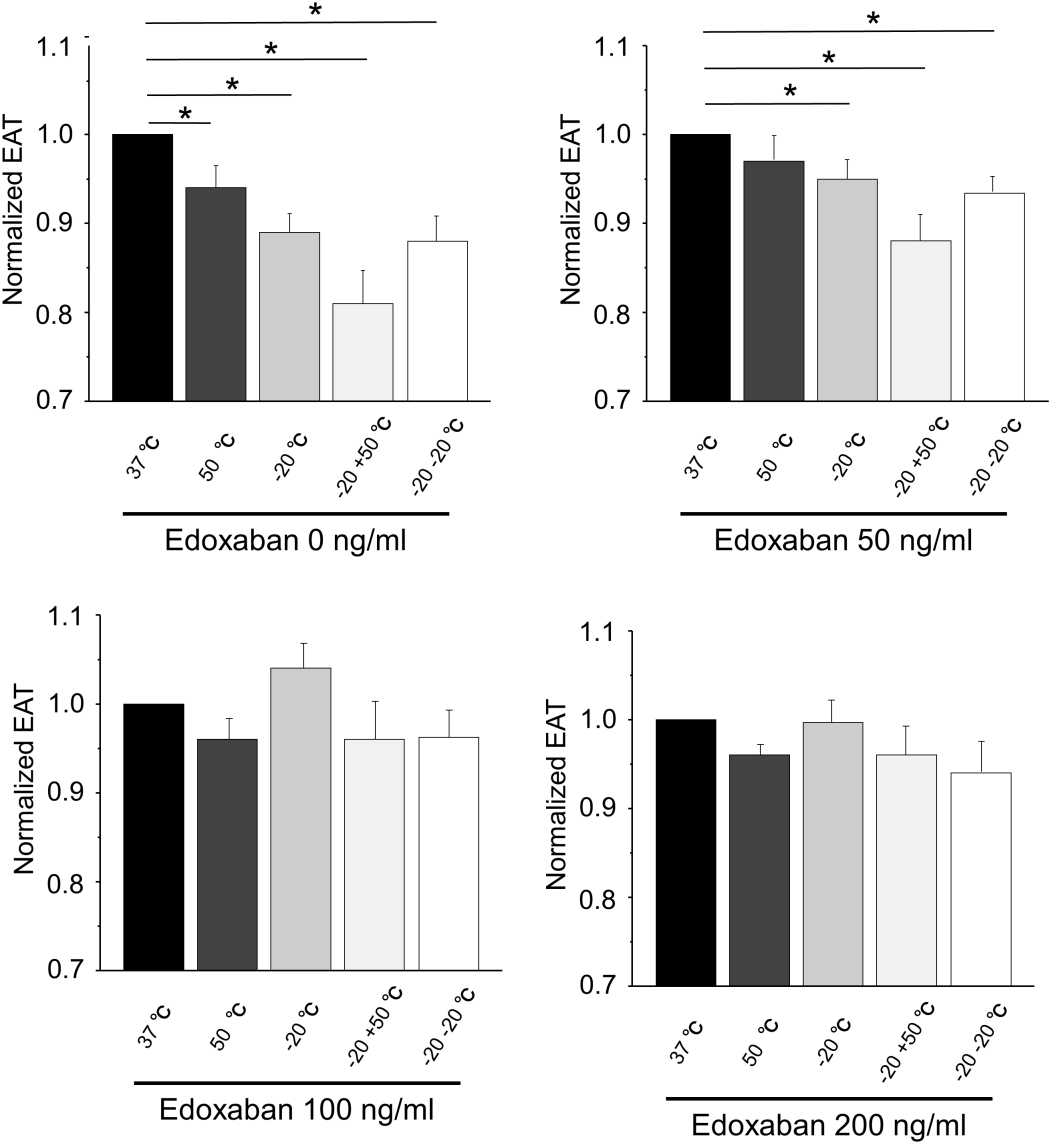
Effect of edoxaban on hypercoagulability induced by transient temperature changes. Edoxaban (50–200 ng/mL) was added to human whole blood with tissue factor, and the end of acceleration time (EAT) was measured after several temperature stimulations. *p < .05 versus control (37°C) by Dunnett test.

Edoxaban prolonged the EAT in whole blood without temperature stimulation in a concentration‐dependent manner (14.1 ± 0.5, 15.6 ± 0.5, 17.1 ± 0.5, and 17.9 ± 0.5 (min) in 0, 50, 100, and 200 (ng/mL), *p* < .001 by ANOVA) (Figure 5). We also evaluated the inhibitory effect of edoxaban on the blood hypercoagulability by temperature stimulations in blood samples with TF. Edoxaban suppressed the hypercoagulability evoked by transient temperature stimulations (Figure 6). The shortening of EAT by temperature stimulations was disappeared by edoxaban at 100 and 200 ng/mL in all conditions of temperature changes.

## 4 DISCUSSION

The risk of embolism increases during the perioperative period of catheter ablation, and one of the causes is the formation of thrombi owing to the activation of the blood coagulation cascade. The blood coagulation is activated during catheter ablation by the insertion of artificial products such as sheaths and catheters or by tissue damage caused by ablation. In this study, as an additional mechanism of coagulation activation during catheter ablation, we demonstrated that the transient temperature changes, which mimics radiofrequency ablation and cryoablation, induced hypercoagulability of whole blood using a novel coagulometer which can sensitively detect changes in blood coagulation status. In particular, the blood coagulability was enhanced under the condition that a trace amount of tissue factor was applied in the blood sample stimulated with the low temperature. In addition, the direct oral factor Xa inhibitor, edoxaban, significantly suppressed the hypercoagulability induced by heat‐ and cryostimulations.

Mild hypothermia (~35°C) had no effect on any part of the coagulation cascade. Moderate hypothermia (<35°C) induced mild platelet dysfunction and decrease in platelet count. And severe hypothermia (down to 12°C) led to a progressive delay in the initiation of thrombus formation. Regarding the effect of lower temperature (~4°C), it was studied the impact of temperatures at the time of centrifugation for plasma preparation on clotting times, and prothrombin time and activated partial thromboplastin time were prolonged in plasma centrifuged at 4°C.^19^ Another report has shown that there was no significant difference in the clotting times when samples were stored at room temperature or on ice.^20^ For longer storage in cooled condition, storage of blood sample in 4°C shortened the prothrombin time, accompanied with increased activity of factor VII.^21^, ^22^ As far as we investigated, however, it has not been elucidated how the sub‐zero degree hypothermia affect the blood coagulation.

This is the first study to examine the impacts of transient drastic temperature changes (50°C and −20°C) applied to whole blood immediately before measuring the coagulability. Moreover, it should be noted that this study used a novel device with a high sensitivity to detect slight changes in coagulability, which might not be detected by ordinary coagulation tests. We clearly demonstrated that the cryostimulation to the blood induced hypercoagulability, especially in condition with TF. It is a novel finding in this study.

Hypercoagulability evoked by transient temperature change stimulations to blood may reflect the coagulation activation during catheter ablation. Okishige et al.^5^ have also reported that the coagulation system was activated by freezing stimulation during cryoablation in pigs. They have inserted a cryocatheter into the right atrium of pigs, applied just only freezing stimulation without causing tissue damage, and then collected blood from the right atrium to measure thrombin antithrombin complex. As a result, thrombin–antithrombin complex was significantly increased after cryostimulation, indicating the local activation of the blood coagulation by low temperature in the atrium. It was reported that the storage of blood sample in 4°C induced “cold activation” resulting in the shortening of prothrombin time, because of the activation of factor VII.^21^, ^22^ This mechanism may explain our findings that cryostimulation induced whole blood hypercoagulability. We found that the sequential stimulation (cryostimulation followed by heatstimulation) tended to enhance the shortening of EAT than repeated cryostimulation. This finding might indicate that cryostimulation and heatstimulation had some additive effect.

We previously reported that DBCM could assess whole blood coagulability, and the EAT was the indicator for global blood coagulability.^16^ In addition, we reported that the maximum acceleration time (MAT) indicated the activity of factor Xa.^15^ In this study, we also calculated the MAT, which showed similar changes as the EAT after heat‐ and cryostimulations. Administration of edoxaban prolonged more in MAT than EAT as reported in previous report.^15^ We focused on the global blood coagulability, thus chose the EAT as an indicator in this study. This study suggests the importance of periprocedural anticoagulation. Hypercoagulation during catheter ablation might cause stroke, TIA, or asymptomatic cerebral emboli. Thus, hypercoagulability evoked by transient temperature changes should be suppressed with an anticoagulant therapy. In this study, we evaluated the effect of the direct oral factor Xa inhibitor edoxaban. The results showed that edoxaban completely eliminated hypercoagulability by both temperature stimulations at clinical blood concentrations of 100 and 200 ng/mL. Even at the lowest concentration of 50 ng/mL (similar to the trough concentration), the hypercoagulability caused by a single temperature stimulation disappeared. The porcine study has also demonstrated that the intravenous injection of edoxaban rapidly and significantly reduced thrombin antithrombin complex to the control level.^5^ As shown in recent clinical studies,^6^, ^7^, ^8^, ^9^, ^10^, ^11^ to prevent periprocedural thromboembolic events, uninterrupted anticoagulation with the oral anticoagulants and maintenance of an adequate activated clotting time (at least 300 s) by heparin are recommended in the expert consensus statement on catheter ablation of AF.^2^

### 4.1 Limitations

There are several limitations in this study. First, this is the in vitro study with static condition. Thus, the clinical significance should be considered carefully. The result of this study might be applicable for the blood in the pulmonary vein during occlusion by cryoballoon. However, after release of occlusion, the blood flow recovered. Thus, the change in blood coagulability should be weakened after cessation of occlusion. On the other hand, we applied heat‐ or cryostimulations for 1 min, which was shorter than the clinical application of cryoballoon ablation. The clinical implication of this study should be done with consideration of these differences in the experimental condition and clinical settings. So, an additional study may be needed to confirm whether the similar hypercoagulability would occur in the local blood near the sites of catheter ablation. We believe that the data from the porcine study by Okishige et al.^5^ support this. Second, there may be more optimal conditions of temperature and duration of stimulation to resemble the actual situations of the sites of catheter ablation. Third, it is unclear to what extent hypercoagulability evoked by transient temperature changes contributes to the risk of periprocedural thromboembolic events in AF patients undergoing catheter ablation.

## 5 CONCLUSION

Transient temperature changes evoked hypercoagulability regardless of heat‐ and cryostimulation in human whole blood. Edoxaban at 100 ng/mL or more eliminated this temperature‐stimulated hypercoagulability.

## FUNDING INFORMATION

This study was supported in part by the Grant‐in‐Aid for Scientific Research from the Ministry of Education, Culture, Sports, Science, and Technology (MEXT) of Japan (No. 16K09494 to T.S.). This work was supported by Daiichi Sankyo Co., Ltd.

## CONFLICT OF INTEREST STATEMENT

TS: Research grant from Boehinger Ingelheim.

## ETHICS APPROVAL STATEMENT

This study was approved by the ethics committee of Tokyo Medical and Dental University (No. 2000–1849 and No.2000–1534).

## PATIENT CONSENT STATEMENT

Written informed consent was obtained from the all subjects participated in this study. Blood samples were collected after obtaining written informed consent.

## PERMISSION TO REPRODUCE MATERIAL FROM OTHER SOURCES

Not applicable.

## CLINICAL TRIAL REGISTRATION

Not applicable.

## DECLARATIONS

*Approval of the research protocol*: This study was approved by the ethics committee of Tokyo Medical and Dental University (No. 2000–1849 and No. 2000–1534). *Informed Consent*: Written informed consent was obtained from the all subjects participated in this study. Blood samples were collected after obtaining written informed consent.

## Data Availability

Available upon request.
